# Manganese toxicity with ephedrone abuse manifesting as parkinsonism: a case report

**DOI:** 10.1186/1752-1947-6-52

**Published:** 2012-02-07

**Authors:** Mudassir Iqbal, Thomas Monaghan, Janice Redmond

**Affiliations:** 1 Department of Neurology, St James's Hospital, Dublin, Ireland

## Abstract

**Introduction:**

Neurologic consequences of manganese toxicity have been recognized since 1837. A new form of presumed manganese poisoning has been reported in drug-addicted persons from Eastern Europe and the Baltic states who have intravenously injected self-prepared methcathinone hydrochloride (ephedrone), which is synthesized from pseudoephedrine hydrochloride using potassium permanganate as a potent oxidant. This clinical syndrome is under-recognized in Western Europe and there are no reported cases in the literature from Ireland.

**Case presentation:**

We report a 30-year-old Eastern European man who presented with a two-year history of gait disturbance. A neurological assessment revealed features of parkinsonism which included hypophonia, hypomimia, mild bradykinesia and rigidity with no resting tremor. He held his arms slightly abducted from his sides when walking, with a reduction in arm swing. Magnetic resonance imaging of his brain showed a high signal on T1 in the globus pallidus and serum manganese levels were raised. He had no response to levodopa.

**Conclusion:**

Manganism secondary to ephedrone abuse causing parkinsonism has emerged in Western Europe in recent years due to mass immigration and often remains unrecognized. This paper highlights the various features of this rare cause of parkinsonism and aids in its recognition and subsequent diagnosis. Neurologists in Western Europe will increasingly encounter such patients.

## Introduction

Neurologic consequences of manganese toxicity have been recognized since 1837 [1]. A new form of presumed manganese poisoning has been reported in drug-addicted persons from Russia, Ukraine and Estonia who have intravenously injected self-prepared methcathinone hydrochloride, which is synthesized from pseudoephedrine hydrochloride using potassium permanganate as a potent oxidant [2,3]. Methcathinone is a stimulant with euphoric effects, known in Russia as ephedrone and by the street names *cat, mul'ka*, and *jeff*. We report a case of manganese toxicity in an Irish hospital where this entity is virtually unknown.

## Case presentation

We report the case of a 30-year-old Eastern European man who presented with a two-year history of gait disturbance. Initial neurological assessment revealed hypophonia, hypomimia, mild bradykinesia and rigidity with no resting tremor. His eye movements were normal. A cognitive and neuropsychological evaluation was unremarkable. His gait showed a slight forward tilt of the trunk from his hips on standing and he held his arms slightly abducted from his sides when walking, with reduction of the arm swing. He tended to walk on the balls of his feet when going forward, seemingly falling forward into the next stride. Moreover, on sitting down from a standing position he deliberately placed himself in front of the seat and gently flexed his knees until he fell backward. He could not walk backward and had frequent falls. His birth and family history were unremarkable.

**Figure 1:**
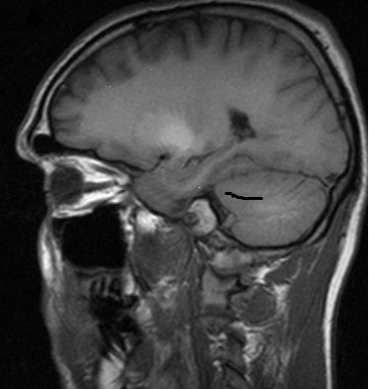
**Brain MRI, T-1 sagittal view showing high signal in globus pallidus**.

Initial investigations included the following laboratory tests: erythrocyte sedimentation rate, autoimmune screen, syphilis serology, vitamin B-12 and folate levels, paraneoplastic screen, copper screen, and serology for hepatitis B and human immunodeficiency virus. All results were normal. Analysis of his cerebrospinal fluid was normal. A chest X-ray and computed tomography brain scan were also unremarkable. However, viral polymerase chain reaction for hepatitis C virus was positive. Magnetic resonance imaging (MRI) of his brain showed increased signal in the globus pallidus on T1 sequences; the signal was normal on T2 and fluid-attenuated inversion recovery sequences (Figure 1). DaTSCAN (ioflupane, 123-I-FP-CIT) imaging was performed in view of his extrapyramidal features and showed normal striatal morphology. He was treated with levodopa and the dose was escalated to nearly 1000 mg/day without any clinical response.

**Figure 2:**
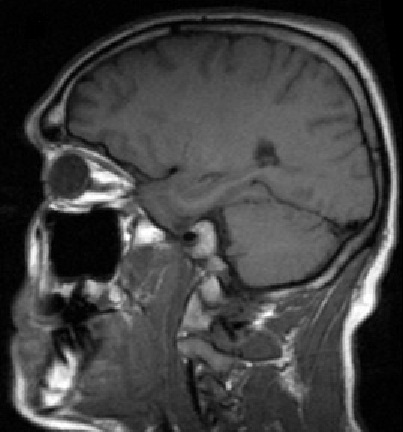
**Brain MRI, T-1 sagittal view shows the high signal has completely resolved two years later**.

Our patient was discharged from hospital with a provisional diagnosis of a possible somatoform disorder as his symptoms could not be explained on an organic basis at the time. He was followed up regularly over the next two years in our neurology outpatient clinic. Serial neurological examinations performed in our clinic over this period remained unchanged. Therefore, a repeat MRI of his brain was performed two years later and showed the high signal on T1 in the globus pallidus had completely resolved in comparison to the previous MRI (Figure 2).

Although our patient denied current illicit drug use, he admitted that he had been using a mixture of heroin, pseudoephedrine (Sudafed), vinegar and potassium permanganate intravenously for two years before presentation. This cocktail was prepared in the home environment and the formula was obtained from the internet. This led us to suspect manganism as the cause for his extrapyramidal features. Serum manganese levels performed two years later were subsequently raised at 573 nmol/L (reference range: 73-218 nmol/L).

## Discussion

The first case of parkinsonism caused by potassium permanganate was reported by Holzgraefe *et al. *in 1986 [4]. In our patient, parkinsonian symptoms had first occurred nine months after poisoning. Manganese is an essential trace metal required for a variety of enzymatic and cellular processes within the human body [5]. Under normal dietary consumption, systemic homeostasis of manganese is maintained by both its rate of transport across enterocytes lining the intestinal wall and by its efficient removal within the liver [6]. The exact mechanism of action of manganese neurotoxicity is unknown. In overexposure to manganese, a high concentration of Mn^3+ ^causes increased auto-oxidation of dopamine in brain regions such as the nuclei of the basal ganglia and leads to redundant generation of free radicals [7].

The pathophysiology of manganese intoxication and Parkinson's disease are both associated with neurological changes in the basal ganglia; the latter correlates with loss of dopaminergic neurons within the nigrostriatal pathway, whereas the former is associated with the preferential degeneration of gamma-aminobutyric acidergic neurons within the globus pallidus.

The strongest evidence indicating the sparing of neurons within the nigrostriatal pathway in manganism comes from studies demonstrating that fluorodopa scans are normal in individuals with high brain manganese levels [8]. Ephedrone abuse is unknown in Ireland and manganism due to ephedrone abuse is often not suspected, primarily because it is uncommon and clinical features are often mistaken for a somatoform disorder. There were several features that led us to suspect manganism in our patient: lack of response to levodopa, hypophonia, absent resting tremor, abnormal 'cock-like' gait, MRI brain scan with a high signal in the globus pallidus on T1 sequences and history of ephedrone abuse. There was no clinical improvement after cessation of ephedrone abuse.

The most important step in the management of manganese toxicity is to prevent it. If removed from the exposure within few weeks or months, the behavioral and neurologic signs often resolve or improve, and can stabilize within two years. In some cases, spontaneous, usually partial, remission has been described several years after initial exposure [9]. However, if the exposure continues, the syndrome becomes irreversible.

## Conclusion

Manganism secondary to ephedrone abuse causing parkinsonism has emerged in Western Europe in recent years due to mass immigration. It should be suspected in cases of atypical parkinsonism and the diagnosis can be confirmed by measuring serum manganese levels in the correct clinical context.

## Consent

Written informed consent was obtained from the patient for publication of this case report and any accompanying images. A copy of the written consent is available for review by the Editor-in-Chief of this journal.

## Competing interests

The authors declare that they have no competing interests.

## Authors' contributions

JR was the consultant neurologist who cared for the patient at the time of presentation and made the diagnosis. MI was the specialist registrar attached to the Neurology unit at the time and is responsible for drafting the case report. TM contributed to the writing of the case report. All authors read and approved the final manuscript.
